# Referral practices of medical practitioners in central South Africa to physiotherapy services for patients living with musculoskeletal conditions

**DOI:** 10.4102/sajp.v77i1.1563

**Published:** 2021-09-30

**Authors:** Roline Y. Barnes, Alida Janse van Rensburg, Jacques E. Raubenheimer

**Affiliations:** 1 Department of Physiotherapy, Faculty of Health Sciences, University of the Free State, Bloemfontein, South Africa; 2 Department of Biostatistics, Faculty of Health Sciences, University of the Free State, Bloemfontein, South Africa; 3 Discipline of Bioinformatics and Digital Health, Faculty of Medicine and Health, University of Sydney, Sydney, Australia

**Keywords:** musculoskeletal conditions, patient referral, physiotherapy, referral practices, burden of disease

## Abstract

**Background:**

Musculoskeletal diseases (MSDs) are a major cause of disability worldwide. It is essential to address effective MSD management, including appropriate referrals to physiotherapists and other healthcare professionals. Limited information is available regarding the referral practices of medical practitioners for patients with MSD. The doctors’ referral practices to physiotherapists can impact the patient population and the South African health system.

**Objectives:**

To investigate or understand the referral practices of medical practitioners in Bloemfontein, South Africa, to physiotherapy services, for individuals living with MSD.

**Method:**

A quantitative study approach, implementing a semi-structured questionnaire, was used. Forty-nine participants completed the questionnaire.

**Results:**

The referral of patients with MSDs by medical practitioners to physiotherapy services varied and multidimensional factors influenced their referral practices. Medical practitioners were unsure of the specific role played by physiotherapists in the management of individuals living with MSD. A need for improved relationships and communication between medical practitioners and physiotherapists was identified.

**Conclusions:**

Medical practitioners regularly referred individuals living with MSD to physiotherapists, but referral practices should be optimised in terms of evidence-based practice and the use of specialised physiotherapy services. In an attempt to decrease the burden of MSD, adequate awareness should be created for improved referral practices between medical practitioners and physiotherapists.

**Clinical implications:**

Collaborative development of detailed guidelines for apt, evidence-based referrals should be developed, to ensure early detection and management of individuals living with MSD. Health care professionals should be educated and encouraged to refer individuals living with MSD to physiotherapists for appropriate management with clinical benefits including improvement of HRQOL and cost effectiveness of this management not only to the individual but also to the health system in South Africa. Physiotherapists should try to communicate their role in the treatment of individuals living with MSD to medical practitioners for the benefit of the patient.

## Introduction

Globally, musculoskeletal disorders (MSDs) form part of the leading group of conditions causing pain and debilitation for individuals, which could lead to their inability to work and impairment of their quality of life (McClatchey 2004; Wang & Wang 2020; World Health Organization [WHO] 2003). MSDs comprise of a variety of conditions with a spectrum of different pathophysiological mechanisms associated anatomically, because of the consequence of pain and physical dysfunction experienced by the individual (Briggs et al. 2018; Woolf & Pfleger 2003).

It is estimated that worldwide, one in three individuals live with a musculoskeletal disorder despite point prevalence varying because of age (Briggs et al. 2018). Over a third of individuals attending a clinic in Cape Town, South Africa, had MSD not because of trauma or previous injury (Parker & Jelsma 2010), which was higher than that reported in community-based studies in the United States of America (USA) (24%) (Muirden 1995), Mexico (17%) (Cardiel & Rojas-Serrano 2002) and the Philippines (16%) (Reginster 2002). Burden of disease profiles are shifting from maternal and nutritional health and communicable diseases to non-communicable diseases, which include MSDs (Briggs et al. 2018). The largest increase in non-communicable diseases is observed in resource-restricted settings (GBD 2016 DALYs and HALE Collaborators 2017; Wang & Wang 2020). Individuals are now living longer with musculoskeletal disorders and chronic diseases, which thus emphasise the importance of focussing on promotive, preventive, and rehabilitative care (Araujo de Carvalho et al. 2017; GBD 2016 DALYs and HALE Collaborators 2017; GBD 2016 Disease and Injury Incidence and Prevalence Collaborators 2017; Wang & Wang 2020). These findings, in association with the global burden of MSD, should make the management of MSDs in South Africa a priority for government and policymakers (Lim et al. 2012).

Furthermore, older individuals are living with multimorbidities of non-communicable diseases, which increase with age and are more prevalent in resource-restricted communities (Barnett et al. 2012; Wang & Wang 2020). Multimorbidity often includes musculoskeletal disorders with musculoskeletal prevalence ranging from one third to more than half of the non-communicable diseases (Duffield et al. 2017). In addition, many risk factors occur in association with other conditions such as, but not limited to, obesity and a sedentary lifestyle (Briggs et al. 2018). A proposed model of care to address the burden of MSD is to involve multidisciplinary teams that could offer integrated person-centred care (Araujo de Carvalho et al. 2017; Keswani, Koenig & Bozic 2016). Depending on the unique needs of the individual living with MSD, the member of the multidisciplinary team who is able to manage the individual best, should be approached first (Cooley 1994). Physiotherapy, as part of the multidisciplinary team, has developed as an essential therapeutic treatment option with defined scientifically based protocols, contributing to important medical and rehabilitation components in the treatment of individuals living with MSD (Odebiyi et al. 2010).

Research has shown that there is a tendency on the side of general practitioners (GPs) not to refer individuals living with musculoskeletal disorders to physiotherapists (Henschke et al. 2014; Rekola et al. 1997; Roberts, Adebajo & Long 2002). Matheny et al. (2000) reported that medical practitioners specialising in family medicine had relatively little confidence in the management of musculoskeletal conditions, including referrals to physiotherapists. This observation was supported by Dennis et al. (2018), who found that patients referred for physiotherapy represented a small proportion of all MSD-related conditions managed by GPs, and that a limited range of problems was considered when referring a patient to a physiotherapist. Archer et al. (2009) also confirmed medical practitioners’ lack of relevant insight regarding the role of physiotherapists for patients living with MSD. The study by Archer et al. (2009) involved medical professionals practising as orthopaedic specialists. They asserted that these specialists had a limited view of the role of physiotherapists. The specialist practitioners believed that the role of physiotherapists was to provide assistive devices and improve patients’ muscle strength. Orthopaedic specialists did not seem to realise the role of physiotherapy to provide individuals living with MSD with coping strategies to deal with the emotional aspects of their disabilities, the improvement of their occupational constraints, or pain management (Archer et al. 2009).

In a systematic review from 14 moderate to low evidence articles, Marks et al. (2017) concluded that physiotherapists can provide more effective management for musculoskeletal disorders than the treatment provided by GPs and orthopaedic surgeons, but the economic impact still has to be investigated.

Based on the literature, it can thus be hypothesised that medical practitioners lack insight and understanding regarding the role of physiotherapy for individuals living with MSD. Therefore, appropriate referrals to a physiotherapist and other healthcare professionals are essential. However, there is limited knowledge regarding medical practitioners’ referral practices of patients living with MSD to physiotherapy services. This lack of knowledge on the subject, which was also identified in South Africa during a comprehensive literature search, may contribute to poor management of MSD and potentially have a negative impact on South Africa’s health system.

A Cochrane review which was conducted in 2016 included 30 studies that assessed a variety of professional interventions by GPs with the intention to improve the management of individuals living with MSD (Tzortziou Brown et al. 2016). The authors concluded that the limited information does not explain the existing practice of patient referrals within clinical pathways, especially for individuals living with MSD. Despite the comment of Freburger, Carey and Holmes (2005) that there is a lack of research investigating the referral practices of medical practitioners to physiotherapists in the USA, no additional research has been done in the last 20 years. These studies therefore emphasised the shortage of research regarding appropriate referrals or referral practices for individuals living with MSD and thus suggested the need for further research in the field of MSD patient referrals.

Several factors complicate the understanding of medical practitioners’ referral practices of MSD patients to physiotherapists. Nigerian (Odebiyi et al. 2010) and Dutch (Kerssens & Groenewegen 1990) studies provide evidence that medical practitioners’ knowledge of physiotherapy influenced their referral practices. A United Kingdom study indicated that medical practitioners’ knowledge of MSD had a greater influence on their referral practices than their knowledge of the health professional to whom they were referring the patient (Kier, George & McCarthy 2013). The personality and/or special interests of the medical practitioner, the sociodemographic characteristics of the patient, the patient’s proximity to a hospital or medical practice and/or the specific nature of the condition, such as the perceived seriousness of the condition (Love et al. 2004), also influenced the referral practices.

Medical practitioners’ management and referral decisions might also be influenced by the circumstances under which they practice (Freburger et al. 2005). Therefore, South African MSD patients’ access and referral to physiotherapy are largely affected by the scarcity of resources and the low medical practitioner-to-patient ratio in low socioeconomic communities (Gcelu & Kalla 2015).

Referral compliance also needs to be considered as an influence on MSD referrals. In a study in the Netherlands, it was found that approximately 12% of patients did not comply with medical practitioners’ referrals for specialist care (Van Dijk et al. 2016). According to these authors, patients living in a demographic area with a lower socioeconomic status practised less compliance with their doctors’ referrals to specialists (Van Dijk et al. 2016). Given the socioeconomic vulnerability of South African patients, referral compliance may be relevant for individuals living with MSD (Jobson 2015).

Studies to identify the current referral system among interdisciplinary team members and the efficiency thereof is important to ensure favourable healthcare for a growing population of individuals living with MSD. Therefore, our study aimed to increase the understanding of interdisciplinary referral practices for individuals living with MSD, specifically between medical practitioners and physiotherapists.

The aim was to identify the physiotherapy referral practices of South African medical practitioners in Bloemfontein, particularly for individuals living with MSD. The objectives of our study were to (i) gain knowledge regarding the referral practices of medical practitioners referring individuals living with MSD to physiotherapy services; and (ii) determine the multidimensional factors that affect the referral practices.

## Methods

### Design and sample selection

Our observational survey study used a questionnaire with both open-ended and multi-choice questions. The respondents could either complete a paper or electronic questionnaire in writing or answer verbally during either an in-person or a telephonic interview with the principal researcher.

The study population consisted of all medical practitioners in Bloemfontein, who were practising in government and private settings at the time of the study and were registered with the Health Professions Council of South Africa (HPCSA). The online register of the HPCSA indicated that 24 674 general medical practitioners had been registered in South Africa in 2016, of which 1156 were practising medicine in the Free State Province. An estimated 452 medical practitioners were situated in Bloemfontein. Because the HPCSA did not provide the contact details for medical practitioners, a search was made to find all publicly available contact information of medical practitioners in Bloemfontein (using search engines such as MedPages, Google and Yellow Pages). In this way, a list of 155 medical practitioners in Bloemfontein was compiled and verified against the HPCSA online register. This list was provided to a biostatistician who initially identified 50 potential participants using Microsoft Excel’s random number generator.

After a poor response rate, 37 more potential participants were selected in the same way from the remaining names on the list, and when this also did not yield a better response rate, a third round of selection of another 20 participants was conducted in the same way. After the third round, we had approached more than two thirds of the doctors on our list with only 50 consenting to participate, although one had not consulted patients living with MSD. The final sample size of 49 was viewed as a sufficient size to allow for generalisation of results for the referral practices of GPs in Bloemfontein, with a margin of error of ± 13% for the most conservative estimate of variance (based on a 0.5 probability).

South African qualified medical practitioners were only included if they managed and referred patients living with MSD to physiotherapists. Those who were unwilling to participate or did not give informed consent were excluded. The medical practitioners who did not consult individuals living with MSD and those who received their tertiary education outside South Africa were also excluded.

### Data collection

A questionnaire was regarded as the most practical way to collect a large amount of information on physiotherapy referral practices for individuals living with MSD. The questionnaire was compiled from relevant research studies (Archer et al. 2009; Clemence & Seamark 2003; Kooijman, Swinkels & Van Dijk 2013; Odebiyi et al. 2010). It was then assessed by a panel of four experts (three academic physiotherapists and one medical practitioner) to ensure that the questions met all the essential requirements and warranted the scientific value of the questionnaire. These experts were identified based on recognition and credibility as experts in the field of physiotherapy by their peers. The questionnaire was improved based on their feedback and was then finalised. The questionnaire consisted of 16 items (nine multiple choice items and seven open-ended items).

The information document, informed consent form and questionnaire were sent to the selected potential participants for their perusal. The participants were requested to return the signed informed consent to the second author via e-mail or fax. To increase the response rate, three types of participation were offered to the medical practitioners. The first option was to complete the questionnaire independently and return it via e-mail or fax. The second and third options were to take part in a semi-structured interview to complete the same questionnaire, either in person or telephonically.

### Ethical considerations

Ethical clearance to conduct the study was obtained from the Health Sciences Research Ethics Committee (HSREC) of the Faculty of Health Sciences, University of the Free State (reference number ECUFS No. 59/2015). Prior to completion of the questionnaire, an information leaflet and informed consent document were provided to each participant. All data obtained were kept confidential, but anonymity could not be ensured because of some of the structured interviews taking place either telephonically or in person.

### Pilot study

Five medical practitioners practising at Universitas Academic Hospital in Bloemfontein, who regularly consulted with patients living with MSD, were requested to participate in a pilot study to assess the face and content validity and efficiency of the designed questionnaire. The pilot study was also conducted to determine if the questions were understood by the participants and to indicate and correct any oversights or difficulties with the questionnaire or the implementation thereof (Van Teijlingen & Hundley 2001). The pilot study also provided an opportunity to measure the time it took to complete the questionnaire during the different completion options available for participation. No challenges were experienced by the participants of the pilot study regarding the completion of the questionnaire. All the participants in the pilot study indicated that the questions were clear and understandable and that the choice of options to complete the closed-ended questions were suitable. No changes were made to the questionnaire after the completion of the pilot study, and therefore the data of the pilot study were included in the main study results.

### Data analysis

All the information from the completed questionnaires was transferred into an Excel spreadsheet prepared by a biostatistician. To validate that the data were transferred correctly, the process of completing the spreadsheet was performed twice. The two spreadsheets were compared after completion. If data differed on the two spreadsheets, corrections were made based on the audio recording of the telephonic interview and looking at the questionnaire completed by the second author during the interview. The completed documents were sent to the biostatistician for analysis after validation of the collected data.

The biostatistician analysed the categorical data and provided descriptive statistics using frequencies and percentages. The open-ended questions were analysed using a quantitative method. The answers provided by the participation options were similar, which enhanced the trustworthiness and validity of the results.

## Results

Fifty medical practitioners agreed to participate and gave informed consent. One participant indicated that he did not treat individuals with MSD and thus was excluded. The resulting response rate was 46.7%, and the study sample represented approximately 11% of Bloemfontein’s 452 medical practitioners at the time of the study. Table 1 shows a summary of the results. Of the 49 participants who completed the questionnaire, 12 (24.5%) were medical specialists, 17 (34.7%) were GPs, 17 (34.7%) were medical officers, and three (6.1%) were in the process of specialisation in Anaesthesiology and Family Medicine. When asked to indicate how long they had been in practice, many respondents answered by simply indicating that it had been a number of years (or, in once instance, months). For the 22 who did provide an estimate, the years in practice ranged from 1 to 40 years, with a median of 17 years (interquartile range [IQR] 10–30). Three participants had between 34 and 40 years’ experience.

**TABLE 1:** Summary of results (*n* = 49).

Variables	*n*	%
**Field of practice**		
General practitioner	17	34.7
Medical specialist	12	24.5
Medical officer	17	34.7
Specialising (Anaesthesiology and Family Medicine)	3	6.1
**How often do you manage patients with musculoskeletal conditions?**		
Daily	31	63.3
Weekly	4	8.2
Monthly	13	26.5
Yearly	1	2.0
**How often do you refer patients with musculoskeletal conditions for physiotherapy assessment and/or treatment?**		
Daily	11	22.4
Weekly	10	20.4
Monthly	27	55.1
Yearly	1	2.0
**Do you apply any specific criteria and/or guidelines for the referral of musculoskeletal patients to physiotherapy?**		
Yes	29	59.2
No	18	36.7
No response	2	4.1
**Is your MSD patient referral influenced by?**		
Effect of physiotherapy on previous patients	47	95.9
The failure of other treatment options	25	51.0
Open lines of communication with physiotherapists	34	69.4
Information on role of physiotherapists during undergraduate training	22	44.9
Information on role of physiotherapists during postgraduate training	41	83.7
Availability of physiotherapists with interest or experience in MSD (e.g. Master’s degree in Physiotherapy, Sport1, OMT)	21	42.9

*Source:* Janse Van Rensburg, A.M., 2017, *A study to identify the physiotherapy referral practices of South African medical practitioners in Bloemfontein for musculoskeletal patients,* viewed 16 June 2021, from https://scholar.ufs.ac.za/xmlui/handle/11660/7566?show=full

MSD, Musculoskeletal disease.

All the participants indicated that they consulted individuals living with MSD on a regular basis despite their specialisation field. Thirty-one (63.3%) of the participants consulted individuals living with MSD daily. Twenty-seven (55.1%) participants referred individuals living with MSD for physiotherapy on a weekly basis. Four of the five orthopaedic surgeons referred individuals living with MSD daily, whereas the other specialists referred individuals living with MSD weekly or monthly.

With regard to using referral criteria for patients with MSD, 29 (59.2%) did and 18 (36.7%) did not use any referral criteria for individuals with MSD. Two participants did not answer the questionnaire item. Two (4.1%) participants also indicated that they were not provided with any referral criteria for individuals living with MSD during their undergraduate studies. The 29 participants using referral criteria were asked to indicate which criteria they used. Rehabilitation to regain full function or full return to activities of normal daily living (48.3%) and clinical reasoning (37.9%) were the most prevalent referral criteria, followed by specific conditions that required physiotherapy (27.6%) and soft tissue injuries (24.1%). A small number of participants referred patients with chronic pain (13.8%) and loss of mobility (13.8%) or because of mechanical injuries as an alternative treatment option (10.3%), or because it was indicated by the post-operative protocol (10.3%). Less frequent criteria for referral included work- or sport-related injuries and neurological problems (6.9% each); whilst stress-related issues, increased body mass index (BMI), financial reasons, and health-related quality of life were mentioned by only 1 of the 29 respondents each.

Fifteen (83.3%) of the 18 participants who were not using any criteria for referring individuals living with MSD to physiotherapy, mentioned that they relied on an index of suspicion that the patient would benefit from treatment or they would refer upon request from the patient or the patient was not responding to pain medication.

The majority of the participants (*n* = 47; 95.9%) indicated that positive outcomes from previous physiotherapy management of individuals living with MSD influenced their referral practices, whilst 34 (69.4%) participants indicated that they believed that personal communication with physiotherapists influenced their referral practices of individuals living with MSD. The knowledge gained during their undergraduate medical training regarding the skills and the role of physiotherapists in the management of musculoskeletal conditions influenced 22 (44.9%) participants’ referral practices. Twenty-one (42.9%) participants indicated that they preferred to refer a patient to a physiotherapist who showed a special interest or had extensive postgraduate experience (Master’s degree in physiotherapy and/or completed courses such as Sport1, OMT) in musculoskeletal conditions. The data reflected multifaceted influences on the medical practitioner’s referral of individuals living with MSD.

## Discussion

The response rate of 46.7% was notably higher than the expected 9% indicated by Braithwaite et al. (2003) for healthcare professionals participating in surveys. This could be attributed to the participants’ positive response to the three options provided for participation in our study. According to Millar and Dillman (2011), choice of participation method in a study could be viewed as an effective approach to encourage participation.

The majority of the participants (*n* = 31; 63.3%) reported seeing individuals living with MSD on a daily basis, confirming that these patients frequently pursue medical care. The findings also reflect the sizable number of individuals living with MSD in Bloemfontein seeking help for their pain and disabilities. Ingram and Symmons (2018) report that approximately 21% of the adult population in the United Kingdom consults their medical practitioners each year regarding MSD.

More than half of the participants consulted individuals with MSD on a daily basis confirming that there is a significant burden of MSD in South Africa. In order to address this issue, several unique challenges need to be tackled. The South African public health sector spends approximately 11% of the government’s annual total budget, which is 6% higher than the 5% of the gross domestic product (GDP) recommended by the WHO, further reflecting the crisis of the burden of diseases in South Africa (Jobson 2015). Furthermore, a third of individuals attending a clinic in Cape Town, South Africa, was living with MSD, which was higher than previously reported in the literature (Parker & Jelsma 2010). In South Africa, as a low to middle income country, access to tertiary healthcare is limited and management of and rehabilitation of MSD-related disabilities at primary healthcare facilities need to be addressed (Parker & Jelsma 2010). A lack of resources across communities in South Africa and the low medical practitioner to patient ratio, which is 0.8 for every 1000 patients, have huge financial and health consequences (Gcelu & Kalla 2015; Jobson 2015). Therefore, the patients living with MSD should appropriately and timeously be referred to a physiotherapist.

Jørgensen and Olesen (2001) and Kerssens and Groenwagen (1990) concluded that a well-established professional relationship between the physiotherapist and the private practitioner was an important factor for referrals, and that previous experiences regarding referral of individuals with MSD to physiotherapy could influence referral practices. Previous experiences regarding referral of individuals with MSD to physiotherapy did indeed influence the practitioners’ referral practices, with 98.0% of the participants indicating that they referred MSD patients more frequently if other individuals had a positive outcome with physiotherapy management. In medical terms, a positive outcome is the improvement of functional limitations or disabilities, the prevention of illness or injury, and the improvement of patient satisfaction. The authors agree with Sackett et al. (1996), who stated that the medical practitioner referral practices for individuals living with MSD should be based on integration of the practitioner’s expertise and the best available clinical evidence for the management of MSD, rather than feedback received from physiotherapists.

Open lines of communication (telephonic or in-person discussions) with physiotherapists apparently have a positive impact on medical practitioners’ MSD referral practices (Clemence & Seamark 2003). Many participants (*n* = 34; 69.4%) in our study indicated that open lines of communication positively influenced their MSD referral practices, irrespective of how long they had been qualified.

Effective interdisciplinary collaboration involving communication and shared decision-making that enables separate and shared knowledge and skills of healthcare professionals to guide patient care, should not be underestimated (Sinclair, Lingard & Mohabeer 2009). Effective communication leads to improved patient and family outcomes, resulting in high levels of patient and family satisfaction, better symptom control and reduction in costs (Kuziemsky et al. 2009). Each member of the interdisciplinary team plays a vital role in providing quality patient care, and if the structure of the team is hierarchical rather than collaborative, there is a higher possibility for ineffective communication (Cristian & Batmangelich 2014). Each team member should have a clear understanding of the role of each member of the team that could contribute to effective patient management to ensure optimal patient care.

Knowledge regarding the physiotherapists’ skills and the practice of physiotherapy gained during medical practitioners’ undergraduate training has an influence on their referral practices (Odebiyi et al. 2010). In our study, 22 (44.9%) participants indicated that the knowledge that they obtained during their undergraduate training influenced their referral practices. It was noteworthy that some of the comments provided by participants in the open-ended questions showed that they felt that medical practitioners did not have nearly sufficient information regarding physiotherapy as a profession, and that the information they did receive during their undergraduate training was too general. Stanton et al. (1985) argued that the only way to ensure appropriate MSD referral practices was to ensure that medical practitioners have comprehensive insight into the role physiotherapists can play in the management of patients. In order to enhance patient outcomes, it is essential that undergraduate medical students should receive education regarding the role or scope of interdisciplinary team members (Kier et al. 2013).

The results showed that 21 (42.9%) participants preferred to refer patients with MSD to physiotherapists who have a special interest or extensive postgraduate experience in the treatment of MSD conditions, and not necessarily to those physiotherapists who received postgraduate training. It could be argued that medical practitioners value the fact that experienced physiotherapists have applied physiotherapy treatment techniques to produce favourable outcomes over time, which carries more weight than the application of theoretical concepts obtained during postgraduate studies.

A shortcoming of our study was the absence of the demographic differentiation between practitioners in the private versus the public sector and its influence on the referral practices of medical practitioners to physiotherapy. In addition, in stark contrast with many other countries, the unique healthcare system and demographic characteristics of the South African population need to be considered when interpreting health-related research.

There are multifaceted factors influencing medical practitioners’ referral practices. The factors which are not explored in our study were personality traits of medical practitioners and patients’ proximity to a hospital or medical practice. More in-depth research exploring all the factors influencing the referral practices of medical practitioners is necessary to make recommendations to change referral practices to the benefit of all stakeholders.

It is recommended that information and guidelines regarding appropriate referral of patients to different members of the interdisciplinary team be developed by the various professional bodies. This information should be made available to medical practitioners at both under- and postgraduate levels of their studies. The latter should also be accompanied by information regarding the specialised roles of interdisciplinary team members.

## Conclusion

Our findings provided valuable insight regarding the current referral practices of medical practitioners in Bloemfontein when referring patients living with MSD to physiotherapy and the different influences that have an effect on their referral practices. Positive outcomes from previous referrals to physiotherapy, personal communication and knowledge gained during undergraduate medical training regarding physiotherapy services, all played a major role in the referral practices of medical practitioners in Bloemfontein. Extensive postgraduate experience determined to which physiotherapist the referral would go.

Improved management, which includes appropriate referrals of individuals with MSD, is important as it could potentially reduce the debilitating effects of MSD on an individual. Another important contribution of improving appropriate referrals of MSD patients is the decreased cost on the healthcare system in South Africa. There definitely is an opportunity to increase potential referrals of patients by GPs to physiotherapy for individuals living with MSD. Furthermore, the opportunity exists to educate undergraduate medical students, registrars and GPs regarding the range of conditions that could be managed effectively by evidence-based physiotherapy interventions.
